# High Tibial Osteotomy for Varus Deformity of the Knee

**DOI:** 10.5435/JAAOSGlobal-D-21-00141

**Published:** 2021-07-09

**Authors:** Ryan Murray, Philipp W. Winkler, Humza S. Shaikh, Volker Musahl

**Affiliations:** From the Department of Orthopaedic Surgery, University of Pittsburgh Medical Center, Pittsburgh, PA (Dr. Murray, Dr. Winkler, Dr. Shaikh, and Dr. Musahl), and the Department for Orthopaedic Sports Medicine, Klinikum rechts der Isar, Technical University of Munich, Munich, Germany (Dr. Winkler).

## Abstract

High tibial osteotomy is a powerful technique to treat symptomatic varus deformity of the knee and is successful when properly indicated and performed. Indications include varus deformity with medial compartment osteoarthritis, cartilage or meniscus pathology. Several techniques exist to correct symptomatic varus malalignment along with concomitant procedures to restore cartilage or meniscus injuries. Evidence supporting high tibial osteotomy for symptomatic medial compartment pathology exists, which provides a durable solution for joint preservation. This review will discuss the indications, techniques, and outcomes for high tibial osteotomies used in the treatment of symptomatic varus deformity of the knee.

The knee is a complex joint able to withstand mechanical stress during weight bearing and range of motion. The biomechanical function of the joint is reliant on the alignment of the lower extremity. Alterations in the alignment of the limb result in abnormal transmission of forces across intra- and extra-articular structures, predisposing to various joint pathologies.^1,2^ Intra-articular pathology, like cartilage or meniscal injury, can be magnified in the setting of limb malalignment as a result of abnormal loading and increased contact pressures. Luckily, surgical procedures have been developed to address lower limb malalignment in an effort to mitigate the associated detrimental effects. In general, medial compartment overload caused by varus malalignment can be corrected with osteotomies of the proximal tibia, including medial opening wedge (MOW) and lateral closing wedge (LCW) procedures. Valgus malalignment is typically addressed with osteotomies of the distal femur and is beyond the scope of this discussion. These alignment-correcting osteotomies are performed in patients with medial compartment pain and symptoms and are commonly performed in conjunction with articular cartilage or meniscal procedures; however, they can be performed in isolation for management of symptomatic unicompartmental osteoarthritis in the setting of lower extremity deformity. This review will outline the indications, planning, execution, and outcomes of high tibial osteotomy (HTO) for symptomatic medial compartment arthrosis with varus deformity.

## Limb Alignment and Preoperative Planning

**Figure 1:**
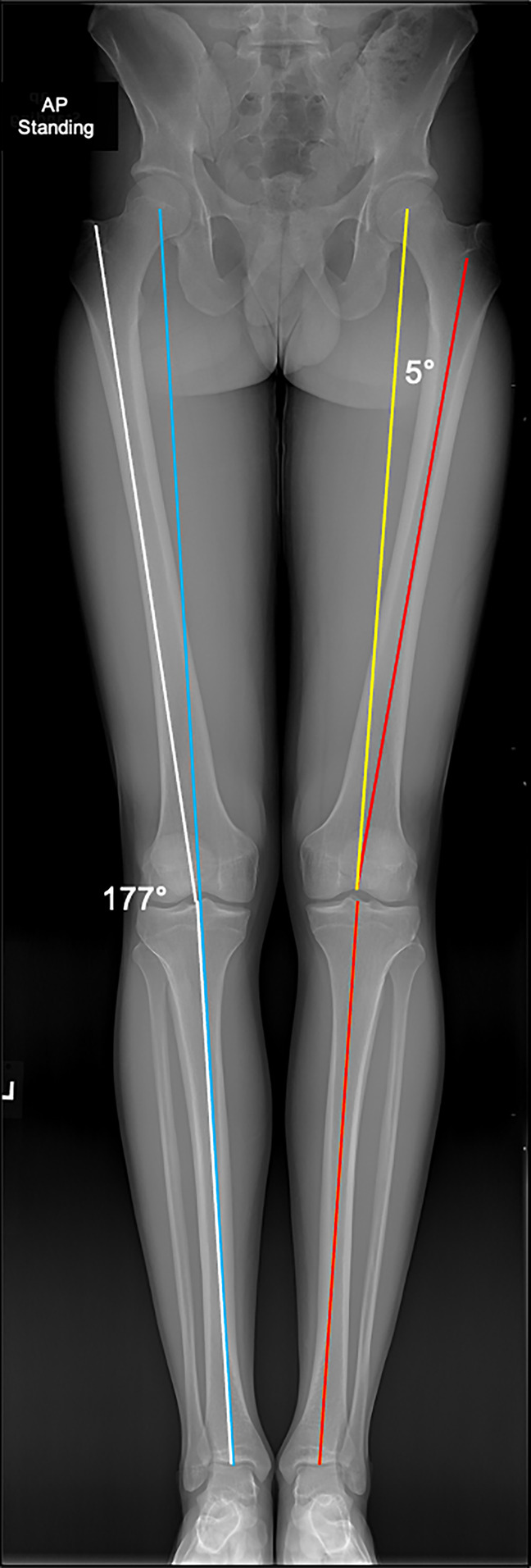
Standing long leg AP radiograph demonstrating the mechanical (yellow) and anatomic (red) axes of the femur and tibia of the left lower extremity. The anatomic mechanical femoral angle is 5°. The mechanical axis of the left lower extremity is marked with a blue line. The anatomic axis of the femur and tibia of the left leg is marked with a white line and forms a 177° anatomic femorotibial angle.

**Figure 2:**
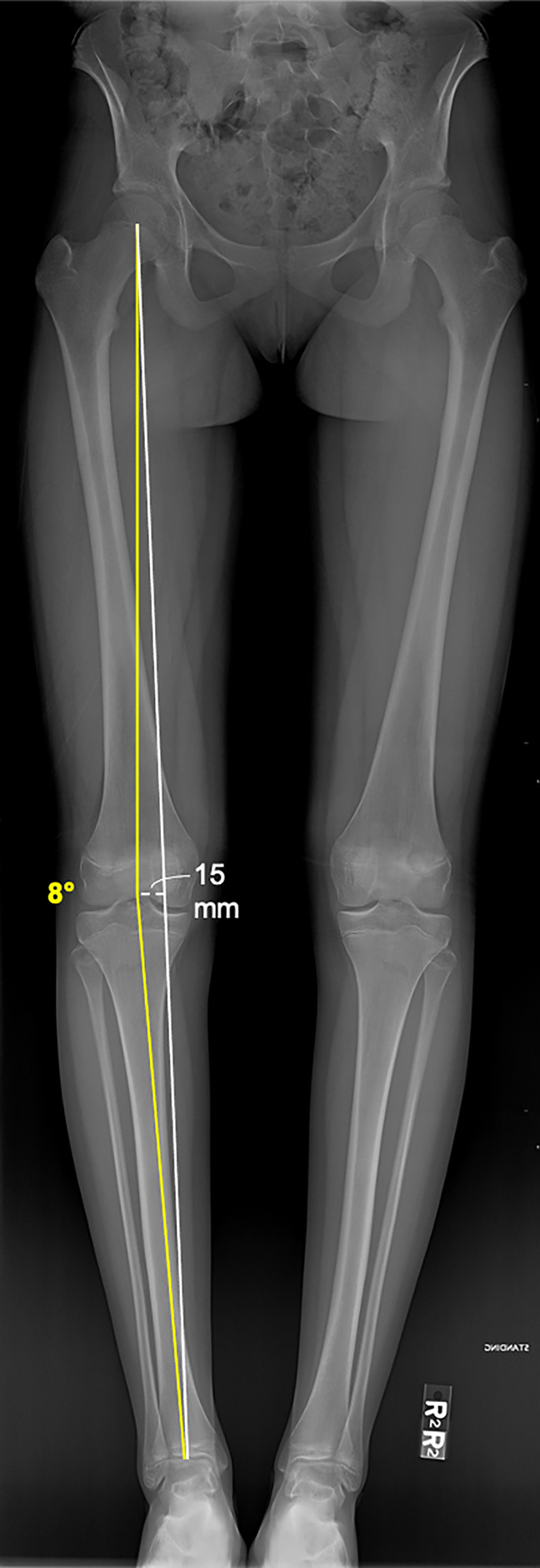
Standing long leg AP radiograph demonstrating varus alignment of the right lower extremity. The femorotibial angle is 8° varus (yellow). The mechanical axis of the right lower extremity (white) is 15 mm medial to the center of the knee joint.

**Figure 3:**
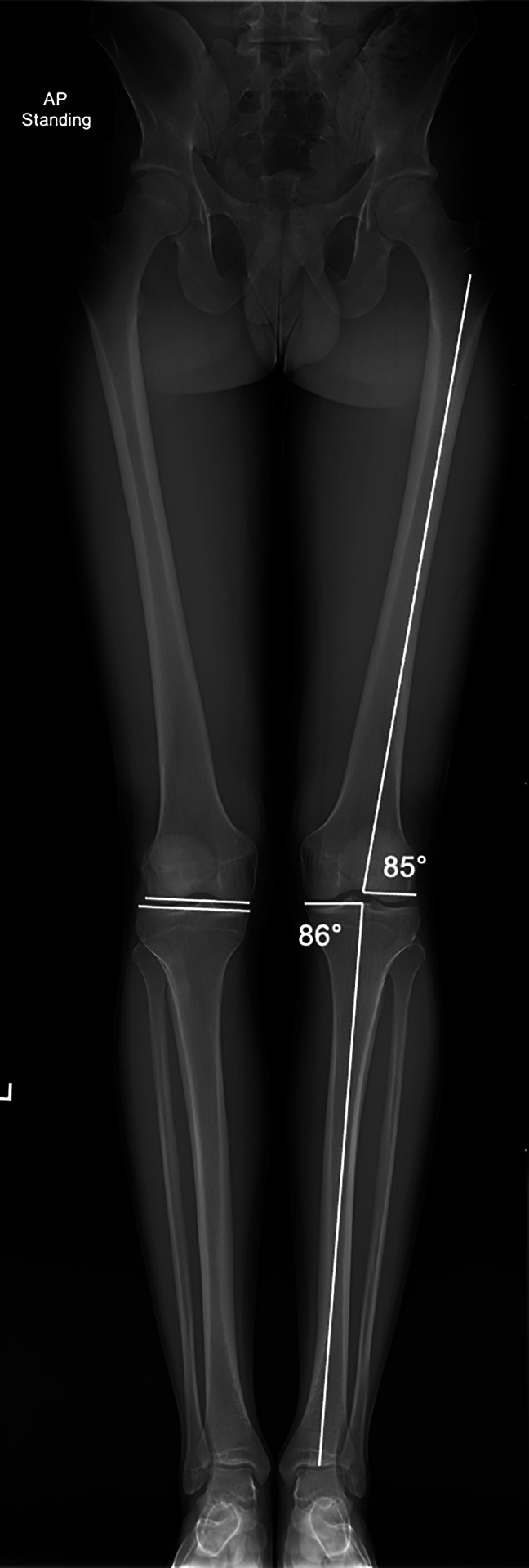
Full-length standing lower extremity radiograph demonstrating the anatomic medial proximal tibial angle of 86° and anatomic lateral distal femoral angle of 85° of the left lower extremity. Femoral and tibial joint lines are demonstrated on the right lower extremity.

Precise knowledge of normal lower limb alignment, the associated anatomical and mechanical axes, and the knee joint orientation angles are of utmost importance for correct surgical decision making. The anatomic femoral and tibial axes represent the respective mid-diaphyseal lines, whereas the mechanical axis of the lower limb connects the centers of the hip and ankle joints^3^ (Figure 1). The horizontal distance between the mechanical lower limb axis and the center of the knee joint, which is measured in the frontal plane, is termed the mechanical axis deviation and is used to quantify frontal plane alignment^3^ (Figure 2). More commonly used to quantify frontal plane alignment is the mechanical femorotibial angle, defined as the angle between the mechanical axes of the femur and tibia^3^ (Figure 1). The mechanical femoral and tibial axes can be determined by connecting the center of the knee joint with the center of the hip and ankle, respectively.^3^ The femoral and tibial joint lines are required to evaluate the joint orientation angles and the joint line convergence angle. The most frequently used joint orientation angles are the medial proximal tibial angle (MPTA) and the lateral distal femoral angle, defined as the angle between the respective joint line and the corresponding mechanical axis^3^ (Figure 3). Physiologic ranges of the main parameters for deformity analysis are listed in Table 1. Furthermore, deformities in the sagittal and axial plane may mimic varus malalignment and therefore require clinical and radiological evaluation.^3^ For this purpose, the most important parameters to be assessed are the posterior tibial slope (PTS) and femoral and tibial torsion.

**Table 1:** Essential Frontal Plane Parameters and Physiologic Ranges

Parameter	Physiologic Range
mFTA (°)	177-181
mMPTA (°)	85-90
mLDFA (°)	85-90
JLCA (°)	0-3
MAD (mm)	3-17 (medial)
PTS (°)	0-15

JLCA = joint line convergence angle (positive values indicate medial convergence), MAD = mechanical axis deviation (positive values indicate medial MAD), mFTA = mechanical femorotibial angle (values >180° indicate valgus alignment; values <180° indicate varus alignment), mLDFA = mechanical lateral distal femoral angle, mMPTA = mechanical medial proximal tibial angle, PTS = posterior tibial slope

The reported physiologic ranges are based on Ref. 3.

In varus malalignment, the mechanical axis of the lower limb crosses the knee joint medially to its center, quantified by an increased medial mechanical axis deviation or a decreased mechanical femorotibial angle (Figure 3). A biomechanical study demonstrated an increased medial meniscal extrusion in varus malalignment, which was furthermore correlated with an increased tibiofemoral contact pressure of the medial compartment.^4^ Accordingly, valgus producing HTOs are primarily performed to unload the medial compartment. Further indications include chronic lateral/posterolateral ligamentous insufficiency or combined with cartilage restoration or meniscus preserving/replacing therapies.^5-7^

Evaluation of the abovementioned parameters is crucial to determine the cause and the location of the underlying varus malalignment and thus to initiate the ideal treatment approach. An HTO is powerful only when applied appropriately to treat varus deformity arising from the tibia itself. However, it has been shown that only 28% of patients with varus malalignment have an isolated tibial deformity.^8^ Moreover, it was demonstrated that isolated HTO is appropriate in only 57% of patients if excessive overcorrection (mechanical MPTA >95°) is to be avoided.^8^ When more significant correction through the tibia is necessary to correct overall lower extremity varus, other surgical options should be considered because postoperative overcorrection of the joint line orientation angles leads to knee joint line obliquity, which is associated with increased intra-articular shear stress and adverse functional outcomes.^9^

## Patient Evaluation and Selection

Nonsmoking patients up to 60 years of age with a high preoperative activity level, a body mass index <30 kg/m^2^, mild to moderate osteoarthritis of the medial compartment defined as Kellgren and Lawrence grade 2 or less, and intact cartilage and meniscus in the lateral compartment are considered the ideal patients for valgus HTO.^10^ Surgical indications include varus alignment with the aforementioned medial compartment pathologies in addition to chronic lateral ligamentous instability and failed anterior cruciate ligament (ACL) reconstruction with deformity in the setting of revision ligamentous reconstruction in a combined or staged fashion when appropriate.

In addition to a thorough history, including past surgical and nonsurgical treatments such as appropriate physical therapy, bracing treatment, and/or injections, along with a complete physical examination, the initial evaluation should include standing alignment radiographs along with dedicated radiographs of the knee. A complete radiographic evaluation for preoperative planning should include weight-bearing AP, PA flexion, and lateral and patellofemoral views. Advanced imaging with an MRI is commonly used to evaluate the extent of cartilage and meniscus pathology in the medial compartment and the integrity of these structures laterally. The constellation of these findings helps to determine the appropriate surgical treatment when nonsurgical treatment has failed to provide adequate symptomatic relief. In cases of varus deformity with a symptomatic medial compartment, HTO may be an appropriate intervention. In some cases, preoperative staging or intraoperative arthroscopy may also be used to determine the extent of medial compartment pathology and ensure the health of the lateral compartment.

When addressing varus malalignment with concomitant ligamentous insufficiency, biomechanical and clinical studies have shown that valgus HTO improves stability in patients with posterior cruciate ligament and/or posterolateral corner insufficiency.^6,11^ However, clear evidence supporting valgus HTO in primary ACL insufficiency with underlying varus malalignment is lacking as clinical studies have not shown higher failure rates after primary ACL reconstruction in patients with varus compared with neutral lower limb alignment.^12^ In contrast, the sagittal alignment represented by the PTS has a biomechanically and clinically proven impact on ACL graft forces and ACL reconstruction failure rates.^1,13,14^ A positive linear correlation between PTS and ACL graft forces was demonstrated,^1^ which is reflected in significantly higher failure rates after ACL reconstruction in patients with increased PTS.^13,14^ Consequently, slope-reducing osteotomies have been recommended in the treatment of multiple failed ACL reconstructions in patients with increased PTS and should be a component of any varus correction in the setting of revision ACL reconstruction with concomitant osteotomy.^15^ Regarding valgus producing HTO, the PTS needs to be assessed preoperatively and monitored intraoperatively to avoid an unwanted or unintended change in the PTS.^16^

## Surgical Techniques

The LCW and MOW techniques in HTO have been shown to be effective in increasing the mechanical MPTA, shifting the mechanical lower limb axis laterally, and thus unloading the medial compartment.^17^ Both techniques have advantages and disadvantages, which are described below.

The LCW technique was originally the standard of care in valgus producing HTO but is becoming increasingly less popular today. However, advantages for the LCW technique, primarily the direct bone-to-bone contact, which is believed to lead to improved bone healing and increased stability of the bone-implant construct, remain and make the LCW technique the preferred one for many surgeons.^18^ Technically, the LCW technique requires an anterolateral surgical approach with meticulous preparation and protection of the common peroneal nerve, followed by a proximal fibular osteotomy or partial fibular head resection to allow subsequent closure of the osteotomy gap.^19,20^ Furthermore, precise preoperative planning is necessary to ensure that an appropriately sized wedge is resected to allow for the desired degree of correction. In addition, when performing the osteotomy, care must be taken to keep a medial cortical hinge intact to avoid disturbances in bone healing and postoperative loss of correction or fragment displacement. After wedge resection and closure of the osteotomy gap, the osteotomy can be fixed using a locking compression plate or bone staples.^19,20^

**Figure 4:**
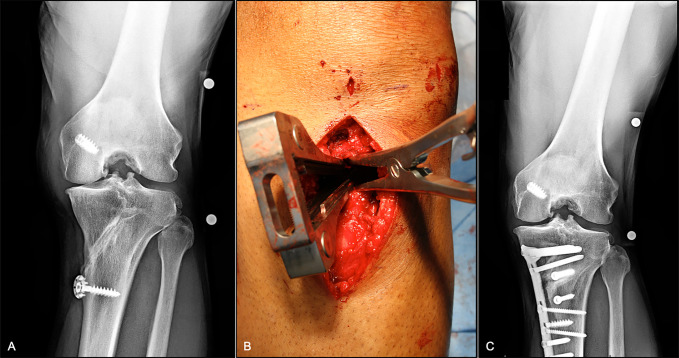
Medial opening wedge high tibial osteotomy. **A**, Preoperative radiographs of a 45-year-old individual with symptomatic medial compartment OA. **B**, Lamina spreaders are used to gently distract the osteotomy once complete. **C**, Two-year follow-up PA flexion weight-bearing radiographs showing preserved medial compartment joint space with mild OA progression. OA = osteoarthritis

The authors' preferred technique is the MOW technique (Figure 4). As such, the patient is positioned in a supine position, with a tourniquet placed on the proximal thigh. The tourniquet is inflated during diagnostic arthroscopy, which is performed to verify intact lateral meniscus and cartilage conditions. After deflation of the tourniquet, an 8- to 10-cm longitudinal anteromedial skin incision, starting at the level of the joint line and directed distally, is made. The patellar tendon, sartorial fascia, and medial collateral ligament are identified through careful dissection. To visualize the tibia, the sartorial fascia is dissected in line with the gracilis tendon, and the tibial attachments of the pes anserine and the medial collateral ligament are mobilized. Approximately 5 cm distal to the medial joint line, two Kirschner wires are placed from distal-medial to proximal-lateral to guide the oscillating saw during the axial osteotomy. In addition, two Kirschner wires (one proximal and one distal to the axial osteotomy plane) are applied in an AP direction, parallel to each other and to the PTS to monitor axial and sagittal plane alignment. First, the frontal osteotomy is performed. Depending on the condition of the patellofemoral joint, the frontal osteotomy is directed either proximally or distally to the attachment of the patellar tendon to avoid unwanted change in the patellar height. After the neurovascular structures have been protected by a Hohmann retractor, the axial osteotomy is performed in 90° of knee flexion aiming for a hinge position in the upper half of the proximal tibiofibular joint about 1 cm medial to the lateral cortical bone.^21^ Osteotomes are used to gradually open the osteotomy gap. At this stage, special care has to be taken to avoid lateral cortical hinge fractures.^21,22^ Modification of the gap ratio or the ratio between anterior and posterior osteotomy gaps enables adjustment of the PTS, whereby preferential opening of the osteotomy anteriorly or posteriorly will increase and decrease the PTS, respectively.^16^ After the desired amount of correction has been verified by intraoperative fluoroscopy, the osteotomy is secured by a locking compression plate, and bone grafting is performed based on surgeon preference and the degree of correction. If concomitant cartilage or meniscus surgery is indicated, the author's preference is to perform these procedures in a single stage using open and arthroscopic approaches as appropriate.

## Postoperative Protocol

For patients undergoing isolated HTO, partial weight bearing for 2 weeks with free range of motion is recommended. Full weight bearing should be achieved after 6 weeks. In the case of an MOW-HTO lateral cortical hinge fracture or a medial hinge fracture with an LCW-HTO, partial weight bearing is extended to 6 weeks postoperatively.^22,23^ Return to recreational sports is permitted after 3 months with a gradual increase in intensity. This postoperative protocol is modified in the presence of any cartilage or meniscus reconstructive procedures and range of motion or weight-bearing restrictions, and return to sport may be delayed relative to the isolated protocol to account for these procedures.

## Outcomes

MOW and LCW-HTO techniques have good clinical and radiographic outcomes when applied correctly with appropriate indications. The longevity of the MOW technique has been illustrated by Hantes et al ^24^ with 95% survival of the native knee joint at 12.3 years in patients less than 45 years old. Schuster et al 5 illustrated that this degree of survival was associated with maintenance of improved IKDC scores over a 10-year time frame. Similar results have been reported with LCW techniques; according to Berruto et al,^25^ 97% of patients reported good-excellent results with a mean survivorship of 12.6 years. However, in a randomized controlled trial by Duivenvoorden et al,^20^ LCW had a higher rate of total knee arthroplasty (TKA) conversion at 6 years compared with MOW, 22% versus 8%. Nevertheless, both techniques have a role based on surgeon preference and training though the MOW-HTO may be a more predictable operation overall.

The LCW-HTO was once the benchmark technique for osteotomy of the proximal tibia; however, modern implant designs have resulted in more widespread use of the MOW technique, which obviates the need for multiple cuts, disruption of the proximal tibia-fibula joint, and dissection of the peroneal nerve, while also not shortening the limb. In considering the MOW-HTO technique, several variables to consider include bone grafting, fixation technique, and weight bearing after surgery. In a randomized controlled trial Fucentese et al^26^ illustrated that iliac crest bone grafting increased the rate of bone healing without a difference in functional outcomes. In terms of fixation techniques, both polyetheretherketone and metal implants are effective in maintaining corrective alignment though metal implants have a higher rate of removal, 46% versus 7%.^27^ Finally, Lansdaal et al^28^ showed that early or delayed weight bearing resulted in no difference in the functional outcome or complication rate.

Concomitant procedures along with an HTO have been increasingly considered though the outcomes are less predictable and more varied owing in part to the complexity of these cases. Harris et al^29^ described a cohort with coronal malalignment, meniscal deficiency, and full-thickness cartilage defects treated with combined osteotomy, meniscal allograft transplantation, and cartilage restoration and showed improvement clinically and based on patient-reported outcomes albeit with a high revision surgery rate of 55.5%. However, the combination of an MOW-HTO with abrasion chondroplasty or microfracture also results in good patient-reported outcomes in cases of medial compartment cartilage loss with fewer complications.^5^ In cases of deep cartilage defects of the medial femoral condyle, osteochondral allograft reconstruction in combination with restoration of neutral alignment by MOW-HTO can result in good patient-reported outcomes and survivorship up to 8.5 years postoperatively.^30^ Deficiency of the ACL is another consideration in the symptomatic varus knee, such that concomitant reconstruction of the ACL along with lower extremity realignment can safely and effectively restore anterior stability and improve functional outcomes.^31^

Because HTO for varus deformity is indicated in the young, active, population, return to work and sport are important considerations. Fortunately, both MOW and LCW techniques allow for high rates of return to both work and sport. In a systematic review and meta-analysis, Kunze et al reported return to various sports and work rates of 76% and 81%, respectively, in a pooled analysis including both techniques. This trend was echoed by Ekhtiari et al,^32^ who illustrated return to sport and work rates of 87% and 85%, respectively, among 1189 patients. In addition, 90% of patients who returned to work did so within 1 year. Furthermore, HTO with combined osteochondral allograft reconstruction for focal medial femoral condyle lesions resulted in a 79% return to sport at less than 1 year postoperatively; however, only 42% of those patients returned at the same level.^33^ Therefore, appropriate education and expectation management is necessary, especially in the young athlete; however, predictable return to sporting activities is possible. The most common alternative to HTO is medial unicompartmental knee arthroplasty, which has been show in some studies to be equally effective in restoring patients' activity levels.^34^ However, in contrast to unicompartmental knee arthroplasty, HTO allows for faster return to impact work and sporting activities with a higher rate.^35^ Thus, HTO is an important technique to consider in candidates who wish to return to sports, especially those involving impact loading.

Finally, as with many joint preservation procedures, the impact on subsequent joint replacement is a consideration. Despite encouraging results and survivorship of HTO, these young patients will oftentimes require a TKA later in life. Fortunately, with modern arthroplasty techniques, 10-year survivorship of cemented TKA was 97%, 90%, and 85% free from aseptic loosening, any revision, and any revision surgery, respectively.^36^ In addition, a systematic review illustrated no difference in TKA outcomes after MOW or LCW techniques; however, more technical issues were reported after LCW-HTO.^37^ Therefore, patients undergoing HTO for varus malalignment can successfully undergo TKA later in life while enjoying the benefits of native joint preservation in their youth.

## Complications

A variety of complications have been reported after HTO, with some of the complications depending exclusively on the technique used (MOW versus LCW). In general, the most commonly reported complications are cortical hinge fractures (29.4%), symptomatic implant, loss of correction (10%), surgical site infections (2%), nonunion or delayed union (1.2%), and peroneal nerve injury (6%).^20,38^ Three different types of lateral cortical hinge fractures are described in MOW-HTO.^22^ Depending on the fracture type, different complications have been reported. Takeuchi type 1 fractures (extension of the osteotomy gap just proximal or within the proximal tibiofibular joint) are most frequently observed with a certain subtype of type 1 fractures defined by a fracture of the posterior cortex being associated with increasing PTS during follow-up.^39^ Takeuchi type 2 (distally directed fracture reaching the distal part of the proximal tibiofibular joint) and type 3 (hinge fracture reaches lateral tibial plateau) fractures are considered unstable fractures and are associated with nonunion/delayed unions and loss of correction.^23,40^ In LCW-HTO, special attention is required in the dissection of the peroneal nerve because peroneal nerve palsy is the most frequently observed neurovascular complication associated with the LCW technique (6%).^20^ Besides surgical dissection, peroneal nerve damage may also occur during fibular osteotomy or secondarily because of increased compartment pressure or inadequate hemostasis. Therefore, a meticulous technique and vigilant postoperative observation are necessary to avoid and detect a potential compartment syndrome.

## Summary

Osteotomies about the knee are useful, sometimes forgotten, procedures that have proven success with the correction of malalignment in the setting of early degenerative changes, ligament reconstruction procedures, and augmentation of joint-preserving procedures such as osteochondral and meniscus reconstruction. Precise preoperative planning is crucial to determining appropriate correction. However, with careful planning and execution, osteotomies and concomitant procedures allow for return to work and sporting activities without limiting future arthroplasty procedures.
